# Towards More Sustainable Food Systems—14 Lessons Learned

**DOI:** 10.3390/ijerph17114005

**Published:** 2020-06-04

**Authors:** Sigrid Kusch-Brandt

**Affiliations:** 1 Civil, Environmental and Architectural Engineering, University of Padua, 35131 Padua, Italy; mail@sigrid-kusch.eu; 2 Water and Environmental Engineering Group, University of Southampton, Southampton SO16 7QF, UK

**Keywords:** sustainability in the food sector, food supply chains, food insecurity, food waste and loss, innovation and change, food governance

## Abstract

Food production, processing, distribution and consumption are among the major contributors to global environmental change. At the same time, food systems need to effectively respond to the demands of a growing world population, and already today many communities and individuals are affected by food insecurity. Moving towards sustainable food value chains is one of the greatest and most complex challenges of this century. To explore promising solutions and specific problems in this context, and to discuss achieved progress, this Special Issue of the International Journal of Environmental Research and Public Health was initiated. The publications enrich our knowledge about essential changes required in the food systems, such as more effective food distribution, avoidance or valorisation of food waste and less meat consumption. Knowing what to change and knowing how to actually achieve such change are two different themes. It becomes evident that there is still an incomplete picture regarding how innovations in the food system can be strengthened to catalyse transformations at a larger scale. Grassroot initiatives require more supporting efforts to effectively influence policies, and the lack of coordination among civil society initiates must be overcome. Sustainability-oriented companies in food supply chains also have a major role to play.

## 1. Introduction

Sustainable food systems are a key challenge for all future development. This Special Issue of the International Journal of Environmental Research and Public Health was initiated as a platform to present approaches and findings that contribute towards more sustainable food systems, and to discuss challenges that need to be addressed with priority. The Special Issue’s information website [1] and the invitation to submit a paper asked the following question:

*“How can the value chain of food production, processing, distribution, consumption, and waste management become more sustainable?”*

A total of 14 submissions were positively evaluated and included in the Special Issue. While certainly these publications do not cover all topics relevant in the field of sustainable food production and consumption, each of these contributions adds substance to the large mosaic of changes and efforts required to achieve food systems that are fit to sustain a prosperous and healthy world in the future, with the human population living in dignity and with responsible stewardship over natural and economic systems. The aim of this editorial is to highlight one key insight from each publication, thus compiling 14 lessons learned from the authors of this Special Issue and their work. These 14 lessons learned are a selection only, and a broader spectrum of insights and findings is available in the publications.

## 2. Overview of Publications in the Special Issue ‘Towards More Sustainable Food Systems’

Table 1 compiles an overview of publications included in the Special Issue, and provides information regarding whether they fall into four core thematic clusters identified.

The publications concentrate on four main themes: (1) food security among the local population, (2) potential impacts of changing environments on food production and already observed manifestations of changes, (3) specific measures for reducing adverse environmental impacts related to food consumption, and (4) understanding how to effectively support desirable changes in the food supply systems.

Table 1 shows that most publications address more than one of these four main themes, which is not surprising when considering the complexity of the food systems. Each publication is rich in insights, and only one insight is selected from each of the 14 publications to be highlighted in this editorial.

## 3. Fourteen Lessons Learned

Table 2 provides an overview of the 14 insights extracted from the publications to be presented in this editorial. Drawing from the 14 publications of the Special Issue, and considering the information compiled in Table 1, the 14 insights discussed in the following mainly, although not exclusively, address three main challenges of food sustainability:

1. Food security, including under changing environments (lessons 1 to 5)
2. Spotlights on specific measures that may reduce adverse environmental impacts related to food consumption (lessons 6 to 9)
3. How to effectively support desirable changes in the food supply systems (lessons 10 to 14)

### 3.1. Food Security, Including under Changing Environments (Lessons 1 to 5)

*Lesson 1: Improvement of infrastructure to facilitate distribution of food continues to be of urgent need in overcoming food insecurity in developing countries.*

By looking at the nutrition situation of the urban and rural population in Zanzibar, the exploratory study of Nyangasa et al. [2] contributes to a better understanding of the factors that influence patterns of poor food consumption and food insecurity among different types of households. Severe food insecurity is more likely to occur in larger households with poor food access, and this is not limited to urban areas but also applies to rural households. Interestingly, Nyangasa et al. observed that in Zanzibar, urban households with good food access have a higher chance of acceptable food consumption than rural households with poor food access. According to the researchers, this may be explained by better infrastructure in urban areas, enabling good the accessibility of foods. The authors conclude that improvement of infrastructure is a key priority in enhancing the distribution of food within the rural–urban areas. In addition, different forms of coping strategies are required, particularly in rural areas, such as efficient food storage techniques and home gardening.

*Lesson 2: Food insecurity in local communities and land tenure insecurity are interlinked phenomena; tenure responsive land-use planning is an essential mechanism in improving food security.*

The work of Chigbu et al. [3], conducted in Rwanda, illustrates that there exists a close connection between land-use decisions and food security outcomes. Access to agricultural land is not enough; rather it is the capacity of the land-user to make critical, household-specific decisions that affects the food security of local communities. Such decisions include what to plant, and how to use the land and harvested agricultural products. Imposition of priority crops on farmers is identified as a form of tenure insecurity in the context of land-use, and, according to Chigbu et al., such an approach failed to be food security responsive in Rwanda. The authors introduce the concept of tenure responsive land-use planning, and propose it as a method for food security improvement.

*Lesson 3: In high-income countries, food insecurity continues to be a challenge of significant scale, and it can be alleviated by rescuing uneaten food from large resorts such as convention centres.*

To et al. [4] present a successful food rescue pilot programme, operated as a cooperation between a convention centre in Las Vegas, USA, and a local food bank. In this context, food safety requires high attention, and only surplus food that has never been served to a guest, has not left the kitchen and has maintained a safe temperature gets donated in the programme. Temperature control is required throughout all further transport and distribution through a network of charitable organisations. In the US, where more than 12% of households are food insecure, while at the same time around 30% of all food sold and prepared at retail and consumer levels is wasted, such food rescue efforts of edible food make an important contribution to more sustainable food systems. Positive impacts on health and wellbeing, higher financial independence and thus more opportunity to avoid or end damaging personal living situations, and the reduction of greenhouse gas emissions are some of the positive impacts of successful food re-distribution initiatives.

*Lesson 4: While the positive impacts of organic fertilisers are generally well known, more efforts are required to understand in detail how climatic change will impact the performance of organic fertilisers.*

Application rates of chemical fertilisers are of high environmental concern, and the use of organic fertilisers, such as livestock manure, is an effective strategy to ensure more responsible nutrient management for agricultural crops and long-term productivity of agricultural soils. However, the experimental results of Khaitov et al. [5] suggest that temperature variations, in particular an increase of the average temperature during the agricultural plants’ growth season (as must be expected in many regions as a result of global climatic change), can significantly impact the performance of organic manure. Soil-climatic conditions can accelerate the mineralisation processes of the applied organic manures. For chilli pepper fruit, Khaitov et al. found a substantially improved fruit yield under elevated temperatures, but the authors highlight the need to consider specific biological properties of different crops, in order to better understand how changing environmental conditions impact the effectiveness of organic amendments to agricultural land.

*Lesson 5: To halt erosion of traditional culinary knowledge and preserve traditional agro-alimentary systems, as a means to reduce local food insecurity risks in a further globalising and urbanising world, it is essential to understand the multifaceted differences in food production and consumption patterns among rural and urban populations.*

A loss of traditional gastronomical traditions and agro-alimentary systems implies a risk of higher vulnerability to food insecurity for local communities, along with an increased risk of negative environmental impacts such as biodiversity loss. Arnés and Astier [6] look at tortilla production in Mexico, where the traditional cuisine is listed as cultural heritage of humanity by UNESCO, and several institutions work to preserve the traditional food systems. Nevertheless, the field study of Arnés and Astier reveals that ancestral knowledge of tortilla making is eroding. This is most evident in urban areas, but it also applies to rural populations. Even where tortillas continue to be referred to as handmade, their ingredients and making might differ. One reason is the substitution of traditional maize with hybrid improved maize, introduced by large seed companies, causing a lack of availability of native grain, especially in urban centres. Another is the practice of mixing native maize with other components in both urban and rural households and gastronomies. The authors also highlight that in rural areas, the consumption of the traditional type of tortillas is associated with poverty and is perceived as a sign of backwardness, while in urban areas it is a luxury. This demand for traditional tortillas in the peri-urban and urban areas might offer opportunities to strengthen traditional practices.

### 3.2. Spotlights on Specific Measures that May Reduce Adverse Environmental Impacts Related to Food Consumption (Lessons 6 to 9)

*Lesson 6: Where food waste cannot be avoided, the coupling of food waste valorisation and wastewater treatment, in an integrated system, creates important synergies to reduce greenhouse gas emissions and deliver additional marketable outputs in a biorefinery approach.*

Around one third of food is never consumed, which wastes resources and generates greenhouse gas emissions. Where food waste cannot be avoided, its valorisation is of high priority. An established food waste valorisation pathway is anaerobic digestion with biogas production. A more environmentally beneficial food waste valorisation pathway is studied by Cecchi and Cavinato [7], namely the simultaneous treatment of food waste and wastewater, which offers several benefits, such as the more effective purification of wastewater in the biological treatment step, a higher biogas yield in the anaerobic digestion step, the recovery of phosphorous, and the option to recover biodegradable polymers in a biorefinery approach as additional marketable outputs. The authors report results from two full scale plants operated in Italy with success.

*Lesson 7: Foods with insect ingredients have high environmental advantages compared to common meat-based foods, but environmental concerns do not have an impact on consumer’s purchase intention, while food neophobia has a significant role in limiting the purchase of insect food.*

Compared to common livestock breeding, raising edible insects emits far less greenhouse gas and consumes significantly fewer resources, such as water and land. Thus, insect food is considered an environmentally favourable choice. However, by conducting a questionnaire survey, Chang et al. [8] found that environmental concerns do not have an influence on a consumer’s decision in favour or against buying food which contains insect ingredients. Arguments that focus on environmental implications therefore are not likely to increase market uptake or support market success. The researchers identified that food neophobia has significant effects on purchase intentions. Consequently, facilitating positive feelings about experiences with edible insect foods can be considered a promising strategy for encouraging more widespread uptake of insect-based food.

*Lesson 8: Consuming less meat has the potential to make a significant contribution to reducing greenhouse gas emissions, but environmental concerns do not have a major impact on the decision to consume meat; however, there might be regionally differing cultural and economic determinants, which merit more attention in order to understand changing food consumption practices.*

Sanchez-Sabate and Sabaté [9] also explore whether environmental concerns impact the food consumption decisions of consumers, in particular the decision to consume meat, which represents a major contributor to global warming. Sanchez-Sabate and Sabaté implemented a systematic literature review and focused on consumers’ intention to consume less meat, and not necessarily no meat at all. It is interesting to note that the latter, i.e., fully turning to vegetarian or vegan food consumption practices, has actually been studied more frequently than the simple reduction of meat quantities consumed (meat-reducers or flexitarians). The findings of Sanchez-Sabate and Sabaté suggest that environmental concerns are relevant for a small minority of consumers only. Even among people who are environmentally aware, reduction of meat consumption is among the least preferred strategies for alleviating climate change. There might, however, be regionally differing cultural and economic determinants which merit more attention.

*Lesson 9: Purchasing power is the strongest factor in influencing the decision to buy organic food, and thus the price is most important when deciding in favour of organic produce, although individual norms, knowledge about health implications and consideration of animal welfare may also play a role.*

Li R. et al. [10] reviewed the literature regarding consumers’ willingness to pay for organic food in China. They found that several factors can influence the decision to buy organic produce, but the consumer’s purchasing power is of decisive impact. It is therefore most essential that marketers of organic foods consider the price. Other factors, such as health benefits or environmental concerns, can play a role in the consumers’ decision, but even when factoring in such considerations, most consumers will not purchase the organic foods if they are expensive compared to conventional foods that are cheaper and readily available. It should, however, be noted that the authors reviewed only a small number of publications, because research on organic food purchasing in China is at an emerging level, with few results available so far.

### 3.3. How to Effectively Support Desirable Changes in the Food Supply Systems (Lessons 10 to 14)

*Lesson 10: Transformative change towards a more sustainable and just food regime needs to disrupt from the bottom up, where local innovations act within their contextual frame, addressing the challenges that their specific communities are facing and looking at scaling in different ways to effectively challenge conventional top-down solutions.*

To understand how desirable changes can potentially be supported, Pereira et al. [11] use the concept of regime shifts to understand the key drivers and innovation processes of past structural shifts in the food system. The dynamics of past transformations are complex, and, according to the authors, previous regimes manifested as a result of powerful actors. Pereira et al. argue that transformative change towards a more sustainable and just regime needs to disrupt from the bottom up, where local innovations act within their contextual frame, addressing the challenges that specific communities are facing and looking at scaling in different ways, thus disrupting conventional top-down solution.

*Lesson 11: Insufficient connections between food-system-oriented initiatives from civil society and small market actors result in the low impact of such initiatives on strategic policymaking; to effectively intervene in food systems, it is essential to engage in participatory coordination, in order to identify common interests among the different types of initiatives, while respecting that the diversity of models brings benefits to the community.*

But how can initiatives from civil society and small local market actors be strengthened to achieve innovation in the food system? Looking at the numerous initiatives across Rome, Italy, Mazzocchi and Marino [12] highlight that initiatives tend to be insufficiently connected, and their experiences lack integration both horizontally (i.e., among them) and vertically (i.e., with respect to strategic policymaking and influence on governance levels that intervene in food systems). To be effective in achieving change, cooperation between initiatives and stakeholders must be improved and integrated into policymaking. Institutionalisation is an important step, but the authors mention that it is a risk to be too closely associated with one political personality; the duration of electoral mandates may be too short compared to the time required for an effective transition of the food system. To coordinate various initiatives to become more effective in influencing food policies, the researchers report success from a participatory approach, which first grouped the various initiatives into clusters and then targeted the identification of common interests among the clusters, while still respecting that each model brings different benefits to the community.

*Lesson 12: In supply chains, sustainability-oriented practices are initially transferred from single focal companies to other actors in the supply chain; a close and stable cooperation functions as learning environment.*

The case study of Zhuo and Ji [13] analyses supply chain coordination in the pig sector in China under a sustainability lens, in particular looking for pathways that foster sustainability along the whole supply chain. The findings illustrate that good practices are initially transferred from single focal companies to other actors in the supply chain, including to upstream farmers with whom the companies cooperate. Provided that the coordinative relationship is close and stable, the cooperation functions as a learning environment for the gaining of sustainable development capabilities, and thus the initial performance of a few supply chain actors catalyses a process where the entire supply chain becomes more sustainable. The authors therefore recommend the establishment of governmental schemes, that provide financial and technological support to core companies for adopting sustainable practices and embracing innovation and learning processes. In addition, effective supply chain coordination methods can facilitate the adoption of sustainability practices across the supply chain.

*Lesson 13: Trust between farmers and agricultural retailers plays an important role in the farmers’ decisions regarding what agricultural materials to buy, including new types of materials, such as fertilisers derived from circular economy schemes.*

Li L. et al. [14] address cooperation at farm level and report the results of a questionnaire survey conducted in China. According to the observations made, good personal relations and trust contribute to reducing information asymmetry between farmers and retailers, and strongly influence a farmer’s decisions regarding what kind of materials to use. Therefore, the establishment and maintenance of personal relations is critical to promoting the security and sustainability of food systems, including through the acceptance of new types of nutrient inputs produced with emerging technologies under circular economy schemes (such as phosphorous recovered from sewage sludge or food waste).

*Lesson 14: Integrating people’s aversion to loss into the design of environmental policies can create more effective environmental protection schemes.*

Finally, Yang and Wang [15] evaluate the effectiveness of different policies that target the disposal of dead animals on farms in China, and whether they discourage farmers from implementing inappropriate measures which endanger public health and environmental protection, but might be economically advantageous. By means of a questionnaire survey, the researchers explored how dead hogs were handled on farms, and found that punishment policies had a significant impact on inappropriate treatment of dead hogs. Farmers feared police detention time more than financial penalties. Financial loss occurring alongside the loss of an animal, i.e., a dead hog, is highlighted as a major concern for a farmer. Interestingly, the loss aversion theory was fruitful to explain the effectiveness of policies in terms of whether an individual engages in inappropriate solutions. The loss aversion theory postulates that individuals are more sensitive to losses than to returns, thus perceiving a positive and negative deviation from the decision point as being different: one unit of loss is considered more important than one unit of gain (the weight assigned to the loss is higher). Thus, evasion of loss is more important for individuals. The authors suggest integrating people’s aversion to loss into the creation of more effective environmental policy schemes.

## 4. Concluding Remarks

The studies presented in this Special Issue contain much more than the 14 insights highlighted in this editorial. The publications reflect a broad research agenda in the field of food system sustainability, and they document both the progress being made in identifying suitable pathways, and the high complexity of the challenges. This enriches our knowledge of the essential changes required in the food systems. Knowing what a desirable future looks like is an important but insufficient achievement. Initiating and effectively supporting progress towards such a desirable future requires additional knowledge and skills. A richness in insights, regarding how to facilitate desirable changes in order to see them happen in the future, is a strength of this Special Issue. The reader will also find information about further research needs.

## Figures and Tables

**Table 1:** Publications in the Special Issue ‘Towards More Sustainable Food Systems’, listed in the order as they are discussed in this editorial (+++: main focus area of publication; +: integrated topic).

	Title of Publication	Local Food Security	Changed Environments	Reducing Environmental Impacts	Supporting Desirable Changes
1	Exploring food access and sociodemographic correlates of food consumption and food insecurity in Zanzibari households (Nyangasa et al. [2])	+++			+
2	Why tenure responsive land-use planning matters: insights for land use consolidation for food security in Rwanda (Chigbu et al. [3])	+++			+
3	The environmental impact and formation of meals from the pilot year of a Las Vegas convention food rescue program (To et al. [4])	+++		+++	+
4	Impact of organic manure on growth, nutrient content and yield of chilli pepper under various temperature environments (Khaitov et al. [5])	+	+++	+	
5	Handmade comal tortillas in Michoacán: traditional practices along the rural–urban gradient (Arnés and Astier [6])	+	+++		
6	Smart approaches to food waste final disposal (Cecchi and Cavinato [7])			+++	
7	Climate change and consumer’s attitude toward insect food (Chang et al. [8])	+		+++	+
8	Consumer attitudes towards environmental concerns of meat consumption: a systematic review (Sanchez-Sabate and Sabaté [9])			+++	+
9	Consumers’ willingness to pay for organic foods in China: bibliometric review for an emerging literature (Li R. et al. [10])			+++	+
10	Food system transformation: integrating a political–economy and social–ecological approach to regime shifts (Pereira et al. [11])	+	+		+++
11	Rome, a policy without politics: the participatory process for a metropolitan scale food policy (Mazzocchi and Marino [12])	+			+++
12	Toward livestock supply chain sustainability: a case study on supply chain coordination and sustainable development in the pig sector in China (Zhuo and Ji [13])			+	+++
13	Moderating effect of dynamic environment in the relationship between guanxi, trust, and repurchase intention of agricultural materials (Li L. et al. [14])	+		+	+++
14	Evaluation of policies on inappropriate treatment of dead hogs from the perspective of loss aversion (Yang and Wang [15])			+	+++

**Table 2:** Overview of 14 insights extracted from the publications of the Special Issue.

	Lesson Learned	Reference
	Food security, including under changing environments	
Lesson 1	Improvement of infrastructure to facilitate distribution of food continues to be of urgent need in overcoming food insecurity in developing countries.	Nyangasa et al. [2]
Lesson 2	Food insecurity in local communities and land tenure insecurity are interlinked phenomena; tenure responsive land-use planning is an essential mechanism in improving food security.	Chigbu et al. [3]
Lesson 3	In high-income countries, food insecurity continues to be a challenge of significant scale, and it can be alleviated by rescuing uneaten food from large resorts such as convention centres.	To et al. [4]
Lesson 4	While the positive impacts of organic fertilisers are generally well known, more efforts are required to understand in detail how climatic change will impact the performance of organic fertilisers.	Khaitov et al. [5]
Lesson 5	To halt erosion of traditional culinary knowledge and preserve traditional agro-alimentary systems, as a means to reduce local food insecurity risks in a further globalising and urbanising world, it is essential to understand the multifaceted differences in food production and consumption patterns among rural and urban populations.	Arnés and Astier [6]
	Spotlights on specific measures that may reduce adverse environmental impacts	
Lesson 6	Where food waste cannot be avoided, the coupling of food waste valorisation and wastewater treatment, in an integrated system, creates important synergies to reduce greenhouse gas emissions and deliver additional marketable outputs in a biorefinery approach.	Cecchi and Cavinato [7]
Lesson 7	Foods with insect ingredients have high environmental advantages compared to common meat-based foods, but environmental concerns do not have an impact on consumer’s purchase intention, while food neophobia has a significant role in limiting the purchase of insect food.	Chang et al. [8]
Lesson 8	Consuming less meat has the potential to make a significant contribution to reducing greenhouse gas emissions, but environmental concerns do not have a major impact on the decision to consume meat; however, there might be regionally differing cultural and economic determinants, which merit more attention in order to understand changing food consumption practices.	Sanchez-Sabate and Sabaté [9]
Lesson 9	Purchasing power is the strongest factor in influencing the decision to buy organic food, and thus the price is most important when deciding in favour of organic produce, although individual norms, knowledge about health implications and consideration of animal welfare may also play a role.	Li R. et al. [10]
	How to effectively support desirable changes in the food supply systems	
Lesson 10	Transformative change towards a more sustainable and just food regime needs to disrupt from the bottom up, where local innovations act within their contextual frame, addressing the challenges that their specific communities are facing and looking at scaling in different ways to effectively challenge conventional top-down solutions.	Pereira et al. [11]
Lesson 11	Insufficient connections between food-system-oriented initiatives from civil society and small market actors result in the low impact of such initiatives on strategic policymaking; to effectively intervene in food systems, it is essential to engage in participatory coordination, in order to identify common interests among the different types of initiatives, while respecting that the diversity of models brings benefits to the community.	Mazzocchi and Marino [12]
Lesson 12	In supply chains, sustainability-oriented practices are initially transferred from single focal companies to other actors in the supply chain; a close and stable cooperation functions as learning environment.	Zhuo and Ji [13]
Lesson 13	Trust between farmers and agricultural retailers plays an important role in the farmers’ decisions regarding what agricultural materials to buy, including new types of materials, such as fertilisers derived from circular economy schemes.	Li L. et al. [14]
Lesson 14	Integrating people’s aversion to loss into the design of environmental policies can create more effective environmental protection schemes.	Yang and Wang [15]
